# Fully-Automated μMRI Morphometric Phenotyping of the Tc1 Mouse Model of Down Syndrome

**DOI:** 10.1371/journal.pone.0162974

**Published:** 2016-09-22

**Authors:** Nick M. Powell, Marc Modat, M. Jorge Cardoso, Da Ma, Holly E. Holmes, Yichao Yu, James O’Callaghan, Jon O. Cleary, Ben Sinclair, Frances K. Wiseman, Victor L. J. Tybulewicz, Elizabeth M. C. Fisher, Mark F. Lythgoe, Sébastien Ourselin

**Affiliations:** 1 Translational Imaging Group, Centre for Medical Image Computing, University College London, 3rd Floor, Wolfson House, 4 Stephenson Way, London NW1 2HE, United Kingdom; 2 Centre for Advanced Biomedical Imaging, Division of Medicine, University College London, Paul O’Gorman Building, 72 Huntley Street, London WC1E 6DD, United Kingdom; 3 Melbourne Brain Centre Imaging Unit, Department of Anatomy and Neuroscience, University of Melbourne, Parkville, Victoria 3052, Australia; 4 Department of Neurodegenerative Disease, Institute of Neurology, University College, London WC1N 3BG, United Kingdom; 5 The Francis Crick Institute, Mill Hill Laboratory, London NW7 1AA, United Kingdom; 6 Imperial College, London W12 0NN, United Kingdom; IGBMC/ICS, FRANCE

## Abstract

We describe a fully automated pipeline for the morphometric phenotyping of mouse brains from μMRI data, and show its application to the Tc1 mouse model of Down syndrome, to identify new morphological phenotypes in the brain of this first transchromosomic animal carrying human chromosome 21. We incorporate an accessible approach for simultaneously scanning multiple *ex vivo* brains, requiring only a 3D-printed brain holder, and novel image processing steps for their separation and orientation. We employ clinically established multi-atlas techniques–superior to single-atlas methods–together with publicly-available atlas databases for automatic skull-stripping and tissue segmentation, providing high-quality, subject-specific tissue maps. We follow these steps with group-wise registration, structural parcellation and both Voxel- and Tensor-Based Morphometry–advantageous for their ability to highlight morphological differences without the laborious delineation of regions of interest. We show the application of freely available open-source software developed for clinical MRI analysis to mouse brain data: *NiftySeg* for segmentation and *NiftyReg* for registration, and discuss atlases and parameters suitable for the preclinical paradigm. We used this pipeline to compare 29 Tc1 brains with 26 wild-type littermate controls, imaged *ex vivo* at 9.4T. We show an unexpected increase in Tc1 total intracranial volume and, controlling for this, local volume and grey matter density reductions in the Tc1 brain compared to the wild-types, most prominently in the cerebellum, in agreement with human DS and previous histological findings.

## Introduction

Down syndrome (DS) is the most common human genetic cause of intellectual disability, affecting around 0.11% of live births in the UK, manifested by physical and cognitive developmental deficits [1]. DS is caused by trisomy of human chromosome 21 (Hsa21), leading to over-expression of genes encoded on this chromosome. The Tc1 mouse model of DS carries a freely segregating copy of Hsa21 and is functionally trisomic for 75% of Hsa21 genes [2,3]. This aneuploid model recapitulates many features, including cardiac defects, short-term memory impairment, motor deficits and mandible malformation seen in humans and other DS mouse models, such as Ts65Dn and Ts1Cje [4,5]. However, morphology of the Tc1 brain has yet to be fully characterised.

We performed a fully automated morphometric analysis of brains from the Tc1 mouse model of DS, using microscopic magnetic resonance imaging (μMRI) and Voxel- and Tensor-Based Morphometry (V/TBM), powerful statistical techniques used to detect subtle local differences in tissue density and physical volumes between groups [6], enabling non-invasive, hypothesis-free structural investigations covering an entire organ or organism. Morphometry obviates the requirement for the laborious expert delineation of regions of interest (ROIs), which are vulnerable to intra- and inter-rater variability and may miss unexpected changes in unexplored areas [7,8]. It may be used to localise and inform subsequent histology, which may otherwise be too time-consuming to cover an entire brain.

V/TBM enjoy widespread clinical use, with software packages such as SPM (http://www.fil.ion.ucl.ac.uk/spm) and FSL (http://fsl.fmrib.ox.ac.uk/fsl/fslwiki). While several groups have undertaken rigorous preclinical morphometric investigations (for example, [9–14]), there remain many barriers to the general uptake of high-throughput morphometry demanded by phenotyping studies, which draw on a vast number of mouse genetic knock-outs and disease models. One is the time taken to image multiple brains in serial, or the significant investment in custom coil arrangements required for parallel imaging [15]. Another is the lack of a complete and fully automatic image processing pipeline for preclinical V/TBM. Existing mouse brain analysis studies often omit descriptions of all but registration and segmentation, or require disparate tools, manual intervention, or that data be adjusted to conform to human-centric software.

Bock et al. [15] scanned up to 16 mice simultaneously *in vivo* using a 7T 40cm bore and a custom array of birdcage receiver coils. The large-bore, high field-strength scanners required to produce high-resolution images are, however, uncommon, and the numerous intricately-arranged coils may be prohibitive for small laboratories [16]. Brains may alternatively be scanned simultaneously with a single preclinical bore and coil–a more prevalent setup–necessitating subject separation into individual images before processing, a problem unique to preclinical imaging.

We have implemented a parallel-subject imaging technique, requiring only a 3D-printed brain holder. We describe automatic separation of brains from multiple-subject images, their orientation to standard space, and the remaining processing for V/TBM, in a single, cohesive software pipeline. We adopt symmetric, inverse-consistent registration, a technique shown to reduce registration biases in clinical data [17]. Reference atlases in a standard space–such as the increasingly popular Waxholm standard [18]–and containing structural brain images and corresponding anatomical labels, enable automation of several steps, including brain masking, subject-specific tissue classification, and label propagation.

Several single-subject and probabilistic mouse brain atlases exist or are in development [10,19,20]. Multi-subject atlas databases are preferred in the human paradigm, but have only recently been implemented preclinically [21,22]. By encompassing natural morphological variation, multi-atlas label fusion techniques reduce bias and increase accuracy over single-atlas and probabilistic approaches by ranking images based upon local similarity [23–25]. Only three multi-atlas mouse brain databases are presently available ([26–28]); only one of which is *ex vivo* ([27]).

**Fig 1:**
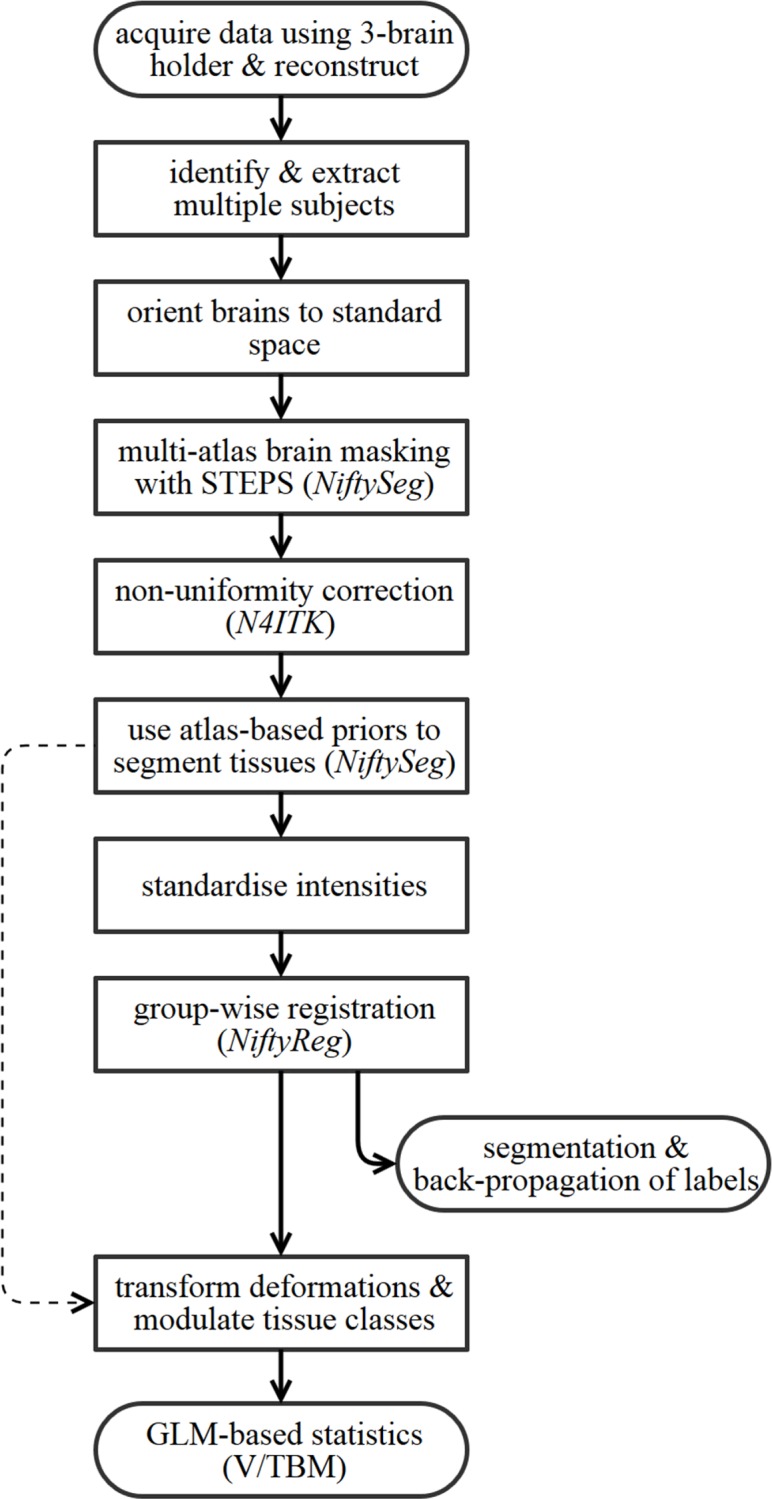
Overview of pipeline steps.

Our pipeline, which we release at http://github.com/nmpowell/mousemorph, is completely automated between scanner, segmentations, and TBM statistical parametric map (Fig 1), and incorporates open-source, freely available cross-platform tools created for clinical data: *NiftyReg* for registration and *NiftySeg* for segmentation (http://cmictig.cs.ucl.ac.uk/research/software). We here show its application to compare 55 *ex vivo* adult mouse brain images of Tc1 and wild-type (WT) littermates, without manual intervention. This first TBM analysis of a DS mouse model reveals global and local volumetric differences–both novel and previously described via histology. We discuss our pipeline and these results and further validate the Tc1 mouse as a preclinical model of human DS.

## Methods

### 2.1. Multiple mouse brain imaging

We scanned two cohorts C_1_, C_2_ of *ex vivo* adult Tc1 and WT brains, and analysed them together to improve statistical power. C_1_ brains were scanned individually. For C_2_ brains, we used a multi-subject protocol.

### 2.1.1. Ethics statement

This study was conducted following approval by the local Ethical Review Process of the MRC National Institute for Medical Research and authorisation by the UK Home Office, Animals (Scientific Procedures) Act 1986 under relevant Project Licence authority. The ERP approved and reported that all work reflects contemporary best practice. High standards in the design and conduct of work were applied and full implementation and consideration of the 3Rs (http://www.nc3rs.org.uk), where appropriate, was made.

### 2.1.2. Mice, genotyping and fixation

C_1_ cohort: 28 male mice aged 4–5 months (14 Tc1, 14 WT littermate matched controls) were taken from a colony maintained by mating Tc(HSA21)1TybEmcf (Tc1) females [3] to F1(129S8×C57BL/6JNimr) males and genotyped as previously described [29]. C_2_ cohort: 28 male mice aged 15 months (15 Tc1, 13 WT littermates) were taken from a colony maintained by mating Tc1 females (from the same colony as those used to breed C_1_) to B6.Cg-Tg(PDGFB-APPSwInd)20Lms/2J males (J20, [30]). All mice included in this study were genotyped negative for the J20 transgene. Mice were genotyped using a polymerase chain reaction assay for the human APP transgene (as Jax stock code 006293). All mice were terminally anaesthetised with an overdose of sodium pentobarbitone, administered via intraperitoneal injection. Brains were perfuse-fixed using an optimised protocol for structural μMRI mouse brain phenotyping [31] and post-fixed (4% formal-buffered saline, 8mM Gd-DTPA) for 9 weeks, then scanned in-skull to prevent damage. One C_2_ WT was excluded from analysis owing to a partially collapsed skull.

### 2.1.3. Image acquisition

Brains were secured with surgical gauze inside a 20ml syringe (C_1_) or within a subject separator (C_2_). Syringes were filled with proton signal-free, non-viscous Fomblin perfluoropolyether (PFS-1, Solvay Solexis SpA., Bollate, Italy) to avoid air interface susceptibility artefacts, and scanned with an Agilent 9.4T VNMRS horizontal bore scanner (Agilent Technologies, Inc., Santa Clara, CA, USA) using an imaging gradient set with a 60mm inner diameter (SGRAD 115/60/HD/S, Agilent Technologies, Inc., Santa Clara, CA, USA). *Single brain protocol (C*_*1*_*)*: 26mm quadrature volume coil (RAPID Biomedical GmbH, Würzburg, Germany), 3hr spoiled 3D gradient echo sequence, with parameters: TE 4.03ms; TR 17ms; flip angle 52°; 6 averages; field of view (FOV) 20.48x13.04x13.04mm (matrix 512x326x326, 40μm^3^ isotropic resolution, as described in detail by Cleary et al., [31]). *3-brain protocol (C*_*2*_*)*: 33mm quadrature birdcage coil (RAPID Biomedical GmbH, Würzburg, Germany); 11hr 4min spoiled 3D gradient echo sequence, with parameters: TE 4.54ms; TR 17ms; flip angle 51°; 6 averages; FOV 32x25x25mm (matrix 800x625x625, 40μm^3^ isotropic resolution).

### 2.1.4. 3-brain subject separator

**Fig 2:**
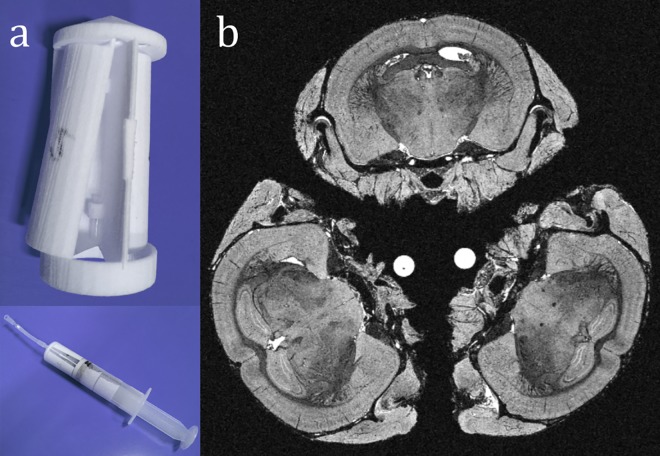
**Multiple-subject scanning**: (a) 3-brain holder and syringe; (b) 3 *ex vivo* mouse brains (1 WT in lower right; 2 Tc1) as they typically appear, in the axial view of a reconstructed μMR image. The holder produces minimal signal. It may rotate within the syringe, so any orientation is possible. The hyperintense dots are identifying agarose markers of different lengths.

We used a custom-designed, 3D-printed plastic mouse brain holder (25.4mm diameter, 44mm length, available from https://figshare.com/articles/CABI_Mouse_Brain_Holder_3_brain_/3394783, doi: 10.6084/m9.figshare.3394783) to secure three skulls inside a 50ml syringe (Fig 2). Others may freely download, modify and print this file for their own use. The 1mm walls precluded touching or partial volume (PV) between neighbouring subjects. This significantly reduced overall preparation time, enabling unsupervised overnight scans and efficient use of all hours of the night (the additional time accounting for increased FOV).

We measured the signal-to-noise ratio, SNR ($\frac{mean\;signal}{standard\;deviation\;noise}$) and contrast-to-noise ratio, CNR ($\frac{signal(GM)-signal(WM)}{standard\;deviation\;noise}$) in both cohorts using the tissue maps created later. Brains were aligned in one z-direction layer to minimise signal drop-off and geometric distortion away from the bore isocentre. To ensure gradient accuracy, the system was calibrated prior to imaging [32]. Gradient linearity was within manufacturer’s limits, as measured within a centred sphere (20mm radius) encompassing the 3-brain FOV. Scaling measurements were performed throughout data acquisition to measure temporal stability. The order of C_1_ scans was randomised, with genotypes interleaved, to avoid the possibility of scanner miscalibration affecting group volume differences. In C_2_, to remove possible group bias caused by remaining distortion or gradient instability, genotypes were mixed randomly within 3-brain scans, and positioning within the 3-brain holder was randomised.

Volumes were linearly scaled to account for phantom-based gradient calibration performed between acquisitions [32]. Scaling factors were C_1_: 1.0321; C_2_: 0.9983 (5 s.f.). T-tests on intra-group mean TIVs did not discern a significant difference between cohorts before (*p*_WT_>0.45; *p*_Tc1_>0.82), or after (*p*_WT_>0.05; *p*_Tc1_>0.14) scaling.

### 2.2. Multiple subject extraction

**Fig 3:**
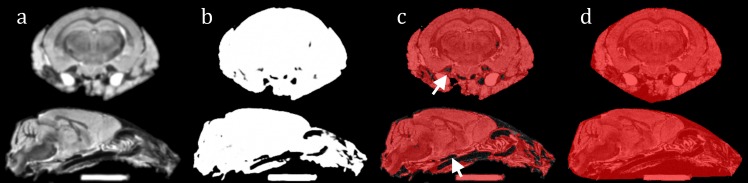
**Extraction steps shown in coronal and sagittal views of a downsampled Tc1 brain image**: (a) smoothed; (b) probabilistic mask after GMM fit; (c) semi-transparent binarised mask of all objects overlaid upon the original image, including agarose marker. Low-signal regions which intrude into the mask, causing incomplete brain coverage, are indicated; (d) convex hull mask.

To robustly extract subjects (Fig 3), isolation via thresholding alone is insufficient [33]: unwanted material may survive. Strong thresholding may discard low-signal tissues, or create concavities where vessels extend into the brain.

We standardised intensities of all acquired volumes (see 2.7 *Intensity standardisation*), smoothed with a 3D Gaussian kernel (FWHM 0.2mm), and used *NiftySeg* to fit histograms with a two-component Gaussian mixture model (GMM, [34]), for subjects and background, omitting prior spatial information. We thresholded the resulting probabilistic image, producing binary masks of all image objects, distinguished using a 3D connected component algorithm with a 6-connected neighbourhood. To ensure complete coverage, we fit a convex hull around each distinct object. This ensured the inclusion of small external features and concavities such as hollow ventricles and the low-signal fissures sometimes present in fixed, *ex vivo* brains. These may include blood vessels; WM tracts such as the optic nerve; and hollows such as the ear canals.

Given the total number of subjects *N* (known *a priori*), we excluded extraneous objects by choosing the *N* objects with the closest volumes to a set of training masks. These were produced by thresholding single-subject images (including skull), and creating a binary convex hull around the largest 6-connected component. The mean mask volume from several such images provided an initialisation for the expected subject volumes. We labelled these *N* objects and used each to crop the original image.

### 2.3. Orientation correction

Brains were aligned to a standard orientation matching that of an atlas, used to propagate brain masks, tissue priors, and parcellations. Skulls may be arranged arbitrarily to fit more into the scanner bore (Fig 2B), or move after placement. The registration algorithms of clinical software, including SPM and *NiftyReg*, cannot reliably resolve the resulting large (>45°) rotations necessary for alignment.

**Fig 4:**
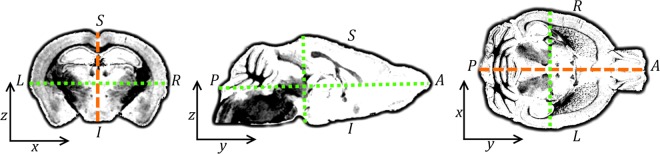
**Illustration of a mouse brain in RAS orientation**: coronal, sagittal, transverse views. Subject +Right, Anterior, Superior parallel to image +*x*, *y*, *z* axes respectively: a “right-handed” orientation common to human atlases [37,38] and Waxholm space [18]. Approximate principal axes (RL, AP, SI) are shown, dotted green and dashed orange. The latter denotes the mid-sagittal plane.

**Fig 5:**
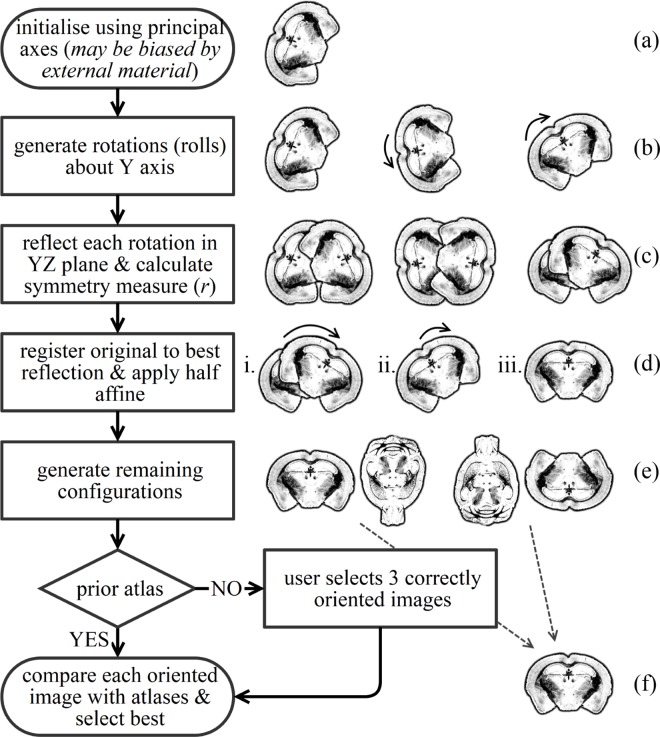
Overview of orientation correction steps, with coronal views. Four configurations are generated (e): in the coronal views, the mouse either faces the viewer or away (L, R interchangeable); in the transverse views, the view is from below or above.

To automate orientation, we exploited the inherent 3D properties of mouse brains. We first assumed that the orthogonal principal axes of mouse brain structural images correspond approximately to their anatomical axes [35] (Fig 4): the antero-posterior (AP), right-left (RL), and inferior-superior (IS) axes, and that AP > {RL, IS}. Skull tissue and other extraneous material (Fig 2B; Fig 3D) may confound this assumption. The image principal axes are the eigenvectors of its inertia matrix [36]. AP thus coincides with the eigenvector with the smallest corresponding eigenvalue. We constructed a rotation matrix using the eigenvectors, and rotated each subject to align AP with the *y* axis (Fig 5A).

Mouse brains exhibit approximate mid-sagittal plane symmetry (the *yz* plane in RAS orientation). We used this feature to correct possible misalignment [39]. To measure symmetry, we reflected images in the mid-sagittal plane and calculated the sample Pearson’s product-moment correlation coefficient *r* between original and reflection (Fig 5B). By composing with additional rotation matrices, we generated 14 rolls about the *y* axis, reflected each, and searched for the optimal rotation which maximised *r* and thus best aligned LR and IS with the *x* and *z* axes respectively (Fig 5C). We corrected remaining misalignments (due to performing a limited number of rotations and tests) by rigidly registering the image pair with the maximum *r*, giving an initial affine transformation matrix, *A*. Applying the (log Euclidean) half of *A* to the original, rotated image thus correctly aligned the anatomical axes (Fig 5D), as Fig 4.

The principal axis eigenvectors omit anatomical direction (AP is equivalent to PA; LR to RL; IS to SI). After correctly aligning principal axes, we composed with 180° rotations (Fig 5E) to generate the remaining candidate orientations in addition to RAS (LAI, LPS, RPI). We registered each to a correctly-aligned image from the University of Florida atlas (UFL, http://brainatlas.mbi.ufl.edu), and selected the candidate with the highest *r* with the atlas as the correct orientation (Fig 5F). A correctly oriented candidate from the four possible final orientations (RAS, LAI, LPS, RPI) could be manually selected for this comparison, instead of using an external atlas image.

### 2.4. Multi-Atlas brain masking (skull-stripping)

Precise brain masks benefit many processing tasks, by restricting the ROI and excluding variable extraneous material. As manual masking is extremely time-consuming at high resolutions, and is susceptible to inter- and intra-rater variability, automated techniques are preferred.

**Fig 6:**
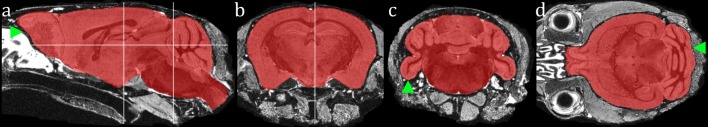
**STEPS brain mask** overlaid on representative slices (a, sagittal; b—c, coronal; d, transverse) of a single WT brain. Solid white lines indicate slice locations. Green markers highlight accuracy, from left, at the olfactory bulbs, paraflocculi, and cerebellum.

To create brain masks, we adopted a multi-atlas technique, employing Similarity and Truth Estimation for Propagated Segmentations (STEPS) label fusion [40], implemented in *NiftySeg*, using *NiftyReg* for affine and non-rigid registration (NRR). We chose the UFL atlas (the only publicly-available multi-atlas database with *ex vivo* images) to best-match our data as a reference, and used parameters recently investigated and optimised by Ma et al. [21] to automatically generate highly accurate masks, including the olfactory bulbs and paraflocculi (sometimes omitted by automated masks–e.g. [41]), and the cerebellum (Fig 6), where external material is in close proximity to the brain and region-growing approaches consequently struggle. This technique produced accurate masks in all 55 brains, without requiring manual correction.

### 2.5. Intensity non-uniformity correction (NUC)

We applied intensity non-uniformity correction within 5-voxel dilated masks using N4ITK [42], found to reliably correct the hardware-induced, low frequency bias field present at high field strengths, using 200 iterations; 256 histogram bins; a 0.15mm FWHM Gaussian kernel to model the bias field; subsampling factor 2.

### 2.6. Tissue segmentation

Brain tissues are segmented to improve mask accuracy, localise analysis to specific classes, calculate total intracranial volume (TIV), and determine changes in local tissue volume or concentration, after registration (VBM). Segmentation via intensity alone, without anatomical priors [13,43], can be unreliable due to morphological variability, PV, intensity inhomogeneity and natural intra-tissue intensity variation. To initialise segmentation, human studies utilise extensive, multi-subject *a priori* tissue probability map (TPM) databases [38], which balance study specificity with naturally-occurring variability. TPMs based on non-representative atlases may misclassify voxels [34]. A comprehensive source of rodent brain TPMs does not yet exist. Sawiak et al. [44] used SPM to create study-specific mouse TPMs, presently the only such public database.

We initialised segmentation of the WT and Tc1 brains using TPMs based upon the National University of Singapore (NUS) atlas [26], preferring its greater number of labels (39) and detail–including cerebellar WM–over the UFL atlas (20 labels). We classified the NUS parcellations as grey matter (GM), white matter (WM), ventricular cerebrospinal fluid (vCSF) or GM/WM mixture, to account for the substantial PV in mouse brains [45], based upon their predominant tissue content (Table 1). We doubled the number of atlas images in the database by reflecting each in the mid-sagittal plane.

**Table 1:** 

**CSF**	cerebral aqueduct, lateral ventricles, third ventricle, fourth ventricle
**GM**	amygdala, auditory cortex, cerebellar cortex, cortex general, dentate gyrus, entorhinal cortex, frontal cortex, general basal ganglia, hippocampus CA1, hippocampus CA3, hippocampus general, hypothalamus, lateral olfactory tract, midbrain (remainder), motor cortex, olfactory system, periaqueductal grey, perirhinal cortex, septum, somatosensory cortex, substantia nigra, visual cortex
**GM/WM mixture**	caudate putamen (striatum), superior & inferior colliculi, thalamus
**WM**	anterior commissure, cerebellar lobules, cerebral peduncle, corpus callosum, fornix system, internal capsule, medulla, optic nerve, pons

Manual binary classification of NUS atlas labels [26]. The pituitary was excluded.

We dilated brain masks by 10 voxels to create a rim region, within which we used a prior-free Expectation Maximisation (EM) algorithm, implemented in *NiftySeg* [34], to classify voxels into 4 classes: background (BG), WM, GM, external CSF (eCSF). This helped to prevent the misclassification of extra-cranial material, meninges and skull as brain tissue, or of cortical surface GM PV voxels as WM [46,47]–a defect of several previously-published mouse brain segmentations.

We registered each atlas image to the data (12 degrees of freedom, DOF, symmetric affine; then symmetric NRR), resampled atlas tissue labels into the data space, and combined them with the rim classes, creating subject-specific probabilistic TPMs reflecting the data’s anatomical variability with 5 classes {GM, WM, eCSF, vCSF, BG}. GM/WM mixture was initialised as 50% GM, 50% WM. The *in vivo* NUS atlas’s large ventricles did not register well to the *ex vivo* data, whose ventricles had almost completely collapsed. Within the vCSF label, therefore, we again used EM to classify voxels as GM, WM or vCSF, and subsequently included this *post-hoc* vCSF result.

**Fig 7:**
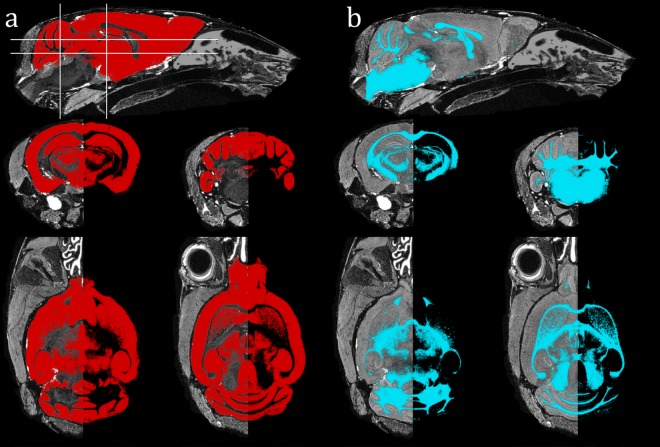
**Probabilistic GM (a; red) and WM (b; blue) tissue segmentations**, in sagittal, coronal and transverse views, overlaid on representative slices of a WT brain. Solid lines indicate slice locations.

These TPMs initialised an iterative EM scheme in *NiftySeg*, spatially constrained with a Markov Random Field (prior strength 0.25). The priors were relaxed (factor 0.25) and regularised (Gaussian kernel standard deviation 0.5mm), to avoid overt atlas bias and account for local anatomical variability [48]. Fig 7 shows resulting segmented tissues. Note external material is excluded, and there is no misclassified WM layer at the cortical surface. The resolution and segmentation-derived tissue probability at each voxel may be multiplied and summed over the entire image to give the total respective volumes of GM, WM and CSF. We calculated TIV, used clinically to account for natural cross-sectional variability of head size [49] as:

$$TIV=voxel\;volume\times \sum_{all\;voxels}\left(P_{GM}+P_{WM}{+P}_{eCSF}{+P}_{vCSF}\right)$$ (1)

Where *P*_*class*_ is the voxel’s segmentation-derived tissue probability, and voxel volume is 40μm^3^. Brain volumes (BV) were calculated as above, excluding CSF.

### 2.7. Intensity standardisation

**Fig 8:**
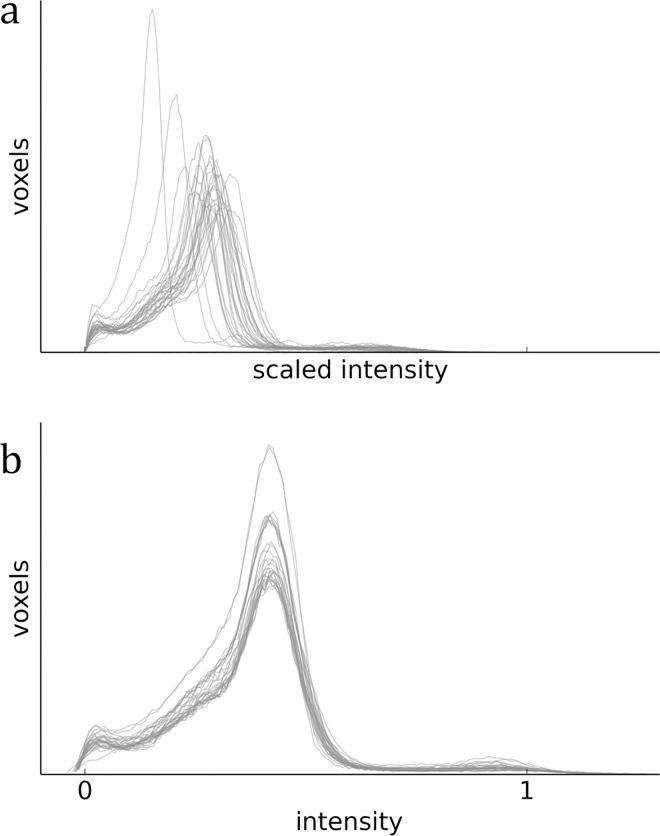
**Intensity standardisation**: (a) original images’ histograms (within dilated masks), scaled to the 0–1 range for comparisson with results of intensity standardisation (b).

An intensity average image is created following each iteration of group-wise registration. We standardised the MR intensity scale beforehand to the approximate 0–1 range, to ensure similar intensity ranges represented equivalent anatomical regions between images, and thus prevent individual images’ noise, intensity extremities and features from dominating this average, which would bias subsequent registrations to particular brains’ anatomy. We used the piecewise linear approach described by Nyúl et al. [50], with 11 histogram landmarks at percentiles *pc* in the configuration *L* = {*pc*_1_,*pc*_99_,*pc*_10_,*pc*_20_,…,*pc*_90_}, to scale intensities between landmark means across images (Fig 8).

### 2.8. Group-wise registration

Images from C_1_ and C_2_ were spatially normalised into common coordinates [9,51] using *NiftyReg*, with 1 iteration symmetric rigid registration, 9 iterations symmetric affine (12 DOF) and 15 iterations symmetric NRR to avoid bias from registration directionality [17,52]. The initial target was randomly selected from the cohort. *NiftyReg* was parameterised with constraints on the final control point spacing (5 voxels), and a penalty term for bending energy (0.005). Normalised mutual information was used as the similarity measure. See S1 File for assessment.

We parcellated the final average image using STEPS and the UFL atlas [21]; the resulting 3D structure labels were used to localise statistical results. We measured the volume of each region via integration of the determinants of the deformations’ Jacobian matrices, at all voxels of the brain [53].

### 2.9. Transformation of deformation fields

For TBM, we took the natural log of the Jacobian determinant *J*_*det*_ at each voxel in the deformation fields. We smoothed these with a 3D Gaussian kernel (FWHM 0.16mm, 4 voxels), chosen to maintain sufficient resolution to identify small features, which helps to account for remaining registration imprecision and renders values more normally distributed, an assumption of subsequent statistical tests [37]. To compare local tissue proportions with VBM, we propagated tissue maps to the group average space and smoothed (as above).

### 2.10. Statistical tests

We generated 3D statistical parametric maps consisting of False Discovery Rate (FDR)-corrected (*q* = 0.05) t-statistics at every voxel of the group-wise registration’s final average image [54]. We performed mass-univariate two-tailed t-tests using the General Linear Model (GLM) on the transformed deformation fields (TBM), and resampled tissue maps (unmodulated VBM) with ANCOVA covariates for animal age, cohort and TIV. The model was thus:

$$Y=b_{1}P(Tc1)+b_{2}P(WT)+b_{3}Age+b_{4}Cohort+b_{5}TIV+\epsilon_{i}$$ (2)

Where the vector *Y* represents, at a particular voxel, the response values from each animal (*log(J*_*det*_*)*); *b* are the regression coefficients; *P(genotype)* is the probability (0 or 1) of each animal being Tc1 or WT; *Cohort* is a binary encoding for C_1_ and C_2_; and *ϵ* is the residual error vector. We controlled for TIV in TBM, to reveal differences in the Tc1 group independent of overall volume. The TIV covariate was excluded for VBM. Levels of the effect of interest (genotype) were compared using contrasts. Tests were constrained to the brain mask to limit the multiple testing problem [55], and to exclude skull and external tissues, which exhibit high inter-individual variability. We also performed two-tailed t-tests on the probabilistic tissue class and parcellation volumes, after normalising to TIV.

## Results

Our analysis revealed several novel features of the Tc1 model, discussed below. Mean (std. dev.) SNR in C_1_ was 29.3 (2.50); C_2_: 13.6 (0.41). CNR in C_1_ was 12.4 (1.40); C_2_: 7.06 (0.40). The sporadic hyperintense rim present in C_1_ was not found to have a deleterious effect upon results (see S2 File). For VBM results, see S4 File.

### 3.1. Global brain volume

**Fig 9:**
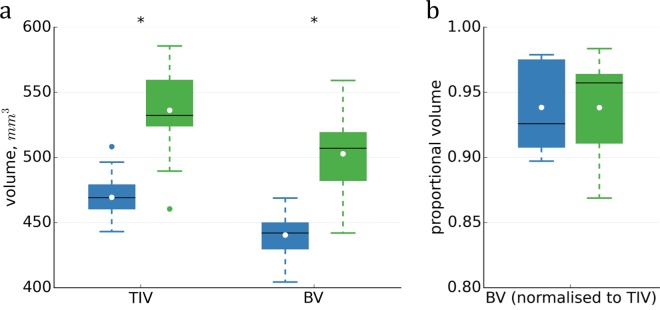
Total intracranial and brain volumes (TIV, BV) of WT (blue) and Tc1 (green). Means (white dots) for absolute volumes (a) were significantly different (*), but after normalisation to TIV (b), BV means were almost identical. Outliers shown are >1.5 IQR.

The Tc1 mice exhibit greater brain and total intracranial volumes than WT littermates (Fig 9; Table 2A), with little overlap. BV was on average 93.8% of both WT and Tc1 TIV (no significant difference, *p* = 0.99), indicating CSF did not play an appreciable role in separating groups.

**Table 2:** 

	WT (N = 26)		Tc1 (N = 29)		*p*	
	mean	std	mean	std	absolute	TIV-normalised
**a**						
GM	316.51	9.63	363.33	21.03	1.82x10^-13^	
WM	123.89	9.52	139.57	11.29	8.86x10^-6^	
BV	440.40	15.48	502.91	28.67	1.10x10^-12^	
vCSF	2.39	0.98	3.43	1.25	0.01	
eCSF	26.70	15.60	29.84	19.18		
TIV	469.49	15.34	536.19	28.33	7.30x10^-14^	
**b**						
amygdala	12.51	1.19	14.55	1.23	1.60x10^-6^	
anterior commissure	1.46	0.13	1.58	0.14		
basal forebrain and septum	13.42	0.49	15.78	0.59	1.15x10^-20^	
brainstem	56.52	1.83	60.08	2.63	9.81x10^-6^	3.39x10^-6^
central GM region	15.32	0.54	17.39	0.65	2.09x10^-16^	
cerebellum	64.21	4.59	61.71	5.60		1.4x10^-11^
corpus callosum & external capsule	17.70	1.19	18.88	1.91		
fimbria	3.55	0.44	4.05	0.49	5.12x10^-3^	
globus pallidus	4.23	0.34	4.57	0.30	5.93x10^-3^	
hippocampus	29.09	1.01	31.44	1.35	4.63x10^-8^	1.06x10^-4^
hypothalamus	12.26	0.49	14.30	0.61	1.24x10^-17^	
inferior colliculus	7.50	0.57	8.12	0.41	4.31x10^-4^	
internal capsule	5.48	0.60	5.40	0.60		1.21x10^-3^
midbrain (remainder)	4.97	0.40	5.72	0.38	4.08x10^-8^	
neocortex	136.02	8.17	145.78	9.68	4.36x10^-3^	2.53x10^-3^
olfactory bulb	26.37	1.85	25.19	1.46		1.26x10^-13^
striatum (caudate putamen)	26.84	1.20	30.72	1.52	4.17x10^-13^	
superior colliculus	9.36	0.62	10.97	0.80	9.52x10^-10^	
thalamus	26.44	0.87	27.61	1.45	0.02	7.85x10^-8^
ventricles	1.65	0.22	2.22	0.45	8.21x10^-6^	4.56x10^-2^

Mean absolute volumes (mm^3^), by group, of (a) probabilistic tissues: BV = GM + WM; TIV = BV + CSF and (b) parcellated regions via integration of Jacobian determinants, and their standard deviations. (Bonferroni-adjusted two-tailed p-values shown, omitted where >>0.05).

Mean absolute volumes of the 20 anatomical parcellations are shown in Table 2B. Most segmented tissues and parcellated regions had greater standard deviation and volume in the Tc1 mice. The brainstem, hippocampus, neocortex, thalamus and ventricles were all significantly larger in the Tc1s, both before and after normalisation. The cerebellum, internal capsule and olfactory bulb were all significantly smaller in the Tc1s after normalisation–but not before. For results from each cohort separately, see S5 File.

### 3.2. Tensor-Based Morphometry

**Fig 10:**
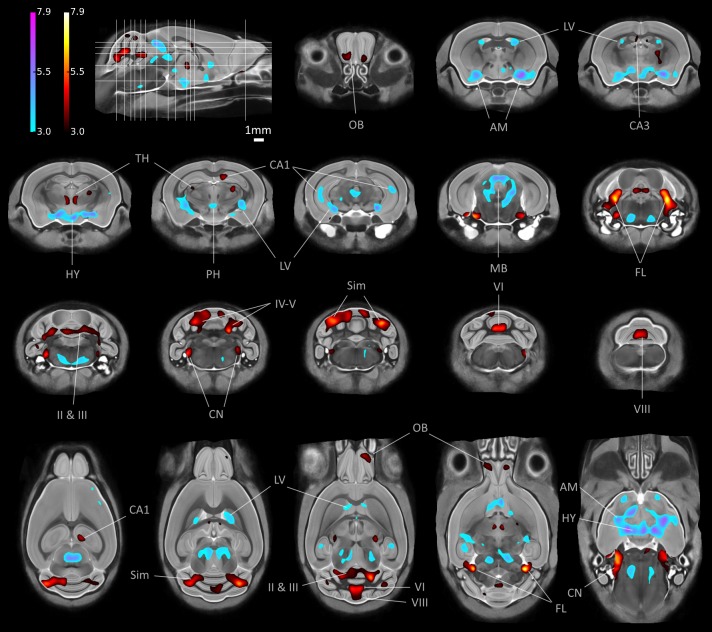
TBM results: FDR-corrected (*q* = 0.05) t-statistics overlaid on slices (locations indicated top left) of the final structural average. Blue: Tc1 group locally statistically significantly larger than WTs; red: Tc1s smaller. AM: amygdala; CA1, CA3: hippocampal sub-regions; CN: cochlear nucleus; FL: flocculus; HY: hypothalamus; LV: lateral ventricles; MB: midbrain; OB: olfactory bulb; PH: posterior hypothalamic nucleus; cerebellar lobules II & III; IV-V (culmen); declive VI and VIII (pyramus); Sim: simple lobule; TH: thalamus.

We used TBM to highlight local volume differences between groups, by including TIV as a confounding factor in our GLM. Fig 10 shows representative slices through the final average image after group-wise registration, with significant voxels overlaid. To localise clusters, we referred to the parcellated labels and Paxinos & Franklin [56].

TBM revealed distinct local volume differences in the Tc1 brains compared with WTs. There were bilateral regions of localised expansion in the amygdala, lateral ventricles and hypothalamus. The reticular nucleus, superior colliculus, and periaqueductal grey regions of the midbrain also showed expansion, possibly secondary to the ventricular aqueduct and 4^th^ ventricle. Unexpectedly, the hippocampus showed a degree of bilateral, localised enlargement in the Tc1 group, in CA1.

We observed significant bilateral reductions in local volume in the olfactory bulbs; the CA3 region of the hippocampus, rostrally; two distinct regions of the thalamus (the rhomboid nucleus and the dorsal sensory-motor region); and in the brainstem, the cochlear nuclei of the medulla. There was a unilateral reduction in the hippocampus medially in CA1. The most prominent volume reductions occurred throughout the Tc1 cerebellum, including bilaterally in the flocculi; the central lobules (II and III) of the cerebellar vermis; the simple lobule and culmen (lobules IV-V); and medially in declive VI and pyramus (VIII). See S3 File for further investigation of significant regions.

## Discussion

### 4.1 Automated pipeline

By enabling computational approaches and, by covering large regions in an unbiased manner, reducing the need for time-consuming, destructive exploratory histology or ROI drawing, μMRI statistical morphometry offers several advantages for phenotyping transgenic and disease model mice. Clinical image processing tools may be used to process preclinical data; however, this requires adjustment to fit human-centric parameters, and manual preparation remains prohibitively time-consuming.

We described a novel multi-subject scanning protocol, and automated methods to eliminate laborious manual processing steps when phenotyping large cohorts of mouse brains. We applied contemporary clinical best-practice techniques (multi-atlas masking, parcellation and segmentation; symmetric registration), without requiring data adjustment, and unified all the processing steps necessary for fully automatic, large-cohort, high-throughput preclinical phenotyping with MRI, from scanner to statistical parametric map. We have tested parameters and image processing steps on over 500 *in vivo* and *ex vivo* mouse brain scans in our lab, ensuring their robustness to variable image quality, shape differences, brains with physical damage or without skulls. TBM results from an *in vivo* study using this pipeline are reported in Holmes et al., [57]. None of the steps failed or required manual intervention for the 55 mouse images described here. Most completed in under an hour using a single modern processor. Group-wise registration is better suited to parallel environments; we used a Sun Grid Engine cluster. Most steps are also applicable to mouse embryo images, provided an appropriate atlas [51].

Atlas-based approaches should be used with caution given the extraordinary number of available mouse strains. A significant issue is compatibility: healthy subject atlases may be poor fits for images exhibiting gross pathology. This concern should be reduced when morphological changes are expected to be subtle, accounted for by registration, where morphometric techniques are more relevant. In the case of gross volumetric differences between groups, or between study data and atlas images, registration parameters may have to be adjusted, or alternative analysis techniques sought. For example, Wong et al. [58] employed intensity differences between aligned mouse embryo images to identify large morphometric changes, such as missing organs. Cardoso et al. [59] showed improvements in parcellation accuracy by propagating labels to severe pathological cases via intermediate images with less severe pathology.

Mouse brain tissue classification is complicated by smaller structures and greater PV proportion than is found in humans. Both may be mitigated using higher field strengths, enabling greater SNR, spatial precision, and contrast [60]. Structural differences between the *in vivo*, skull-stripped NUS atlas used for tissue segmentation and our *ex vivo*, in-skull data were resolved with NRR. Our tissue classifications include fine WM detail, including within the cerebellum and PV regions such as the striatum and midbrain. By employing an atlas, and explicitly modelling background signal, external tissues, PV and CSF, we averted misclassifications which have befallen previously published TPMs, including the presence of a brain-enveloping ‘rim’ where GM PV is misinterpreted as WM, or the corruption of classifications by intensity inhomogeneity.

### 4.2 Morphometry of Tc1 brains

Whereas BV is reduced by around 18% in human DS, and mandible size is also reduced [8,61], we observed significantly increased Tc1 tissue volumes (Fig 9; Table 2A). Using landmarks, O’Doherty et al. [3] measured mandible reduction in Tc1s, but found no overt craniofacial malformation or reduction in skull size. Our consistent, unexpected global finding indicates the utility of whole-brain MR and tissue segmentation over histology and landmark measurements, which are necessarily localised and limited to a few subjects.

All tissues and most parcellated regions displayed greater volume variance in the Tc1s. O’Doherty et al. [3] reported that approximately 66% of cells in the Tc1 brain retain Hsa21. This mosaicism for Hsa21 in the Tc1 strain can vary between organs, mice, and with genetic background, and could lead to phenotypic variation [5]. Olson et al. [62] observed that in human DS, “most DS phenotypes are incompletely penetrant and variable in expressivity–the mechanism(s) by which increased gene dosage causes any specific DS feature is not established”.

Tc1 mice have some rearrangements of Hsa21 [2]: this may contribute to the brain megaly. Pinter et al. [63] suggested greater subcortical GM proportions in DS patients, and preservation of parietal cortex GM volume, may result from insufficient apoptosis.

In humans and mice with deletion or truncation of the Hsa21 gene *DYRK1A* (dual-specificity tyrosine-phosphorylation-regulated kinase 1A), brain size and weight is reduced [64,65]. This gene is thought to be tied to many DS phenotypes, and modulated by the presence of other genes [66]. It is dose-dependent and hence, in humans and Tc1 mice, overexpressed [29]. In two mouse models of partial trisomy, overexpressing the *DYRK1A* gene, Sebrié et al. [64] and Guedj et al. [65] found increased thalamus, midbrain, colliculus and total brain volumes (measured via MRI, weight and histology). In the thalamus, neuronal density and number increased, while neuron size and extracellular space decreased. Conversely, cell density was negatively correlated with *DYRK1A* dosage in the hippocampus and somatosensory and entorhinal cortex. This may underlie our TBM results, which showed expansion of the Tc1 thalamus and midbrain. Guedj et al. [65] noted that Ts65Dn mice, also with three copies of *DYRK1A*, do not exhibit elevated BVs, and that other genes trisomic in the Ts65Dn mouse may compensate.

The ventricles are enlarged in adult human DS [8] and both VBM and TBM detected their bilateral enlargement in the Tc1s. This is likely underestimated in *ex vivo* brains, which shrink slightly during fixation as tissues relax and ventricles partially collapse [12]. The effect is mitigated when skulls are retained [14], and we expect the same systematic effect across groups. Brain expansion due to hydrocephalus has been reported in a DS model [67]; however, these mice died by 10 weeks of age, and the Tc1s did not exhibit gross ventricular enlargement. Overall vCSF volumes differed significantly between groups before, though not after, correction for TIV.

Bianchi et al. [68] recently observed, via histology, impaired neurogenesis in the olfactory bulbs of 13-month-old Ts65Dn mice, and remarked that this may parallel the loss of smell in older human DS individuals. The reductions seen here suggest there may be a similar functional impairment in Tc1 mice.

Cerebellar GM was reduced in a VBM study of non-demented people with DS, and exhibits reduced overall volume compared with TIV in humans [8,69] and the Ts65Dn, Ts1Cje and Tc1 mouse models [3,21,62]. Our TBM analysis reveals that rather than the cerebellum being uniformly reduced in volume, reductions have discrete local foci. There is evidence cerebellar lobules have distinct functional correlates [70]. It may be possible to map local volume reductions to functional topography and hence to behaviour in Tc1 mice.

The cerebellum is associated with fine motor control and cognitive processes. In children with DS, cerebellar hypoplasia is implicated in motor and speech difficulties [63]. Galante et al. [71] found motor learning and coordination deficits in Tc1 mice. Histological staining revealed reduced internal granule layer density in the Tc1 cerebellum compared with WTs, mirroring observations of the Ts65Dn and Ts1Cje models [3,62,72]. We repeated these findings with VBM, showing reduced GM density in the granule cell layer of several lobules (S4 File). This supports the utility of VBM for informing histology. VBM also showed bilateral reductions in GM density in the entorhinal cortex, recapitulating the progressive atrophy of this region in human DS [47].

VBM detected reduced GM in the dentate gyrus (DG) hippocampal region. Long-term potentiation in the DG–synaptic plasticity thought directly related to long-term memory–was reduced in Tc1 mice [3], and behavioural observations demonstrated reduced spatial working memory [73]. We observed bilaterally elevated GM proportion in the CA3 region of the Tc1 hippocampus. Insausti et al. [74] measured elevated neuronal numbers in Ts65Dn CA3, and suggested this may compensate for reductions in DG, although Kurt et al. [75] found normal neuron density, but reduced synapse density, in both structures. Witton et al. [76] also showed decreased synapse density in the DG, and related this to poorer performance of Tc1 mice in a radial arm maze, compared with WTs. These cellular changes could underpin the differences in VBM GM signal observed here.

Morphometry is dependent upon intra-group structural registration accuracy. As registration is intensity gradient-driven, contrast is crucial to the success of morphometry, and a driver of increasing field strengths. Accuracy within homogeneous structures, such as the hypothalamus and brainstem, may be impeded: a local change in the centre of such a structure may be missed by TBM; a uniform change may be compounded and elicited only at its boundary. This may explain why the TIV covariate did not account for some local expansions.

Our 3-brain protocol (C_2_) realised lower SNR and CNR than single-brain (C_1_) scans, likely due to the larger coil required for 3-brain imaging. Kale et al. [77] suggested an SNR of around 20 was optimal for registration accuracy (assessed in S1 File). We included C_1_ to enhance the power of voxel-wise statistical tests. Van Eede et al. [78] found that while false positive rates were below 1%, FDR (*q* = 0.1) recovered only 38% of 20% simulated volume changes, and that TBM generally underestimated volume reduction.

### 4.3 Comparison with parcellation

Employing STEPS-based parcellation, Ma et al. [21] reported shrinkage of the cerebellum and olfactory bulbs, but no other structures, in Tc1 brains relative to BV, which was increased. Controlling for TIV, we repeated these findings using Jacobian integration, and additionally found the internal capsule to be reduced in size, possibly thanks to the increased contrast the group average image provides over individual scans; this thin structure is not easily segmented. Parcellation enables volume- and shape-based analysis of substructures, but is limited in specificity by atlas detail. TBM here localised the contributory regions of difference *within* those structures. Furthermore, we observed local changes within the hippocampus, thalamus and ventricles undiscerned by parcellation. This surrogate biomarker is more useful for informing precise histological follow-up investigations.

Imprecise external atlas registration may have caused slight volume mismeasurement. Currently-available multi-subject atlases (UFL, NUS) are limited by relatively low contrast and resolution compared with those achievable at high field strengths; many opportunities exist for extending their size and quality. A database with more subjects, parcellations, finer detail and greater contrast would aid the specificity of segmentation-based approaches, and could complement V/TBM by more precisely localising significant voxels–for example, within cerebellar nodules. Results should improve further with the increased availability of such atlases.

## Conclusion

We showed a novel phenotyping pipeline’s application to *ex vivo* brains from the Tc1 transchromosomic mouse model of Down syndrome, and identified novel phenotypes, including unexpected overall brain volume increase, and local volume and GM density reductions in the cerebellum, consistent with previous histological findings in this model, and human DS.

Our software shall be made freely available at http://github.com/nmpowell/mousemorph upon publication. The homogenisation of scan parameters, standardisation of analysis pipelines, and improved availability and accuracy of TPMs and atlases enable multi-site, large cohort studies, increasing the feasibility of μMRI and morphometry as important, powerful preclinical phenotyping tools. To aid these efforts, and reproducibility, our dataset, masks and tissue classifications will also be made available online.

## Supporting Information

S1 File Group-wise registration assessment. (DOCX)

S2 File Hyperintense rim. In this supporting text we measure and discuss the implications of an observed “hyperintense rim” in some of the brain images.
(DOCX)

S3 File Effect sizes. This supporting information shows effect sizes at selected individual voxel locations within the TBM results.
(DOCX)

S4 File VBM results. (DOCX)

S5 File Cohort 1 and 2 parcellation volumes. This supporting information includes volumes of segmented tissues and parcellations, as Table 2, from each cohort individually.
(DOCX)
