# Supplementary material for: Fully-Automated μMRI Morphometric Phenotyping of the Tc1 Mouse Model of Down Syndrome
Source: PLoS One. 2016 Sep 22;11(9):e0162974. doi: 10.1371/journal.pone.0162974 (PMC5033246; doi:10.1371/journal.pone.0162974)
Supplement: S1 File — (DOCX) [file pone.0162974.s001.docx]

## S1. Group-wise registration assessment

We quantified the progressive improvement in image alignment at each iteration of group-wise registration (GWR), and justified the number of iterations, by measuring the intensity standard deviation *σ_I_* between resampled images at every voxel within the brain. Inter-image intensities in equivalent aligned regions should have low standard deviation, thanks to standardisation prior to GWR. Fig A shows the mean *σ_I_* over all voxels, at each iteration, within the brain mask, for all brains, and for C_1_ and C_2_. After a dramatic decrease with the first iteration of NRR, *σ_I_* reaches a plateau after 5-10 iterations.

We sought to assess whether the difference in SNR and CNR between cohorts affected the precision of spatial alignment. We resampled each binarised brain mask into the final GWR average space. For each resampled mask, we computed the Jaccard index with every other resampled mask in the cohort, and calculated their mean and standard deviation. We compared cohorts’ mean Jaccard indices with a t-test. There was no appreciable difference (p>0.9; Cohen’s d = 0.0065): GWR performed similarly in aligning brain masks from each cohort.

We also used the per-voxel standard deviation to compare cohorts’ alignment (Fig A). Deviation in voxel intensities should arise from alignment offset, natural variations in local tissue intensity, and noise. Fig A illustrates that after NRR, C_1_ and C_2_ had similar mean standard deviations, over the whole brain.


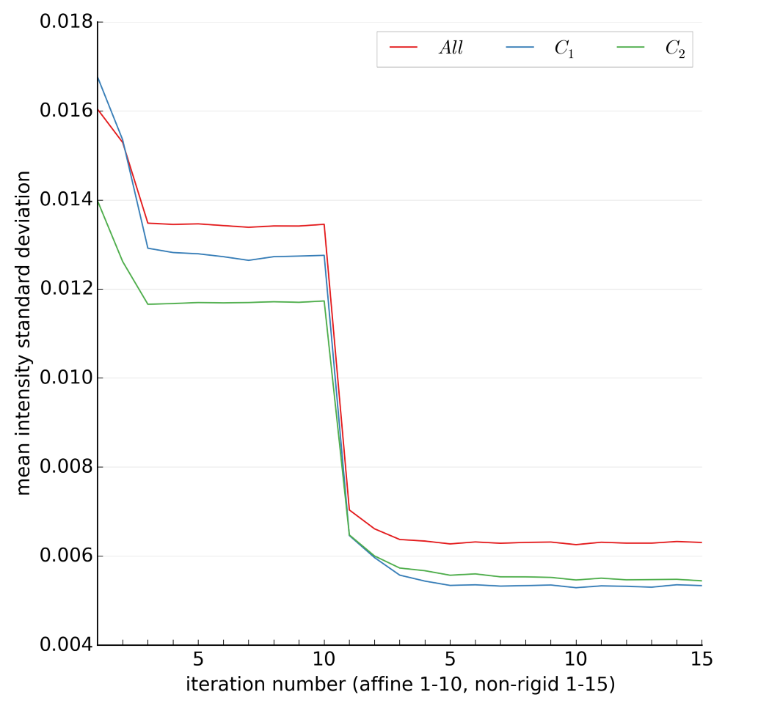


Fig A: **Group-wise registration assessment**: Mean standard deviation of brain voxels in resampled images, within the brain mask, for all brains and each cohort individually.
