# Supplementary material for: Fully-Automated μMRI Morphometric Phenotyping of the Tc1 Mouse Model of Down Syndrome
Source: PLoS One. 2016 Sep 22;11(9):e0162974. doi: 10.1371/journal.pone.0162974 (PMC5033246; doi:10.1371/journal.pone.0162974)
Supplement: S2 File — In this supporting text we measure and discuss the implications of an observed “hyperintense rim” in some of the brain images. (DOCX) [file pone.0162974.s002.docx]

## S2. Hyperintense rim

We noted a hyperintense ‘rim’ partially enveloping most C_1_ brains. Cahill et al. [79] suggested a similar artefact measuring up to 500µm in thickness resulted from improperly dissolved perfusate, or a high perfusion rate, causing blockages and pressure build-up, resulting in ‘bubbles’ of high intensity perfusate forming between meninges and brain tissue, causing compression and hence potentially rendering TBM unreliable. We therefore sought to characterise this artefact in our data.

C_2_ brains were perfuse-fixed in our laboratory using our standard protocol: commercial formal-buffered saline (VWR International Ltd., England), together with a low flow rate (2.5-3 ml/min) [31]. C_2_ brains did not exhibit the artefact. C_1_ brains were perfuse-fixed at a different site, at 3ml/min, where powder PFA was mixed with the same commercial solution.

CSF is also hyperintense in T2* images, and may become trapped during perfusion-fixation. The ‘rim’ volume was taken as the eCSF volume from tissue segmentation. Visual inspection ensured all parts of the rim were included. eCSF occurs naturally, is particularly prominent in models of brain atrophy [80], and pools in the cisterns. We measured its 3D thickness using a Laplacian field-based algorithm typically applied to the cortex [81].

In contrast to Cahill et al. [79], we found a positive correlation between eCSF volume and BV (r=0.449, p=0.017), suggesting the rim in C_1_ did not compress the brain. It was most prominent within the interhemispheric fissure of the cortex, and never completely enveloped the brain. In C_1_, mean (standard deviation) eCSF volume was 44.13µL (8.38µl), 9.53% of average BV. In C_2_: 12.0µL (4.08µL), 2.48% of mean BV, and no eCSF volume exceeded 23.7µL. There was no appreciable correlation between eCSF volume and BV in C_2_ (r=0.02, p=0.92). The mean thickness of the eCSF layer for all C_1_ brains was 162µm (75µm). We only measured thickness where it was greater than 1 voxel (40µm); as the rim was never fully enveloping, the mean over the entire brain surface will thus be much lower. As these measurements reveal the artefact to be less severe, and do not show the correlations reported by Cahill et al. [79], and additionally as we combined C_1_ and C_2_ during group registration, reducing the relative contribution of brains with the artefact to the structural average and to statistics, we do not believe it denigrated V/TBM or volumetric results, or made registration unreliable.

References

1. Cahill LS, Laliberté CL, Ellegood J, Spring S, Gleave JA, Eede MC. van Lerch JP, et al. (2012) Preparation of fixed mouse brains for MRI. NeuroImage 60(2) 933–9. doi: 10.1016/j.neuroimage.2012.01.100
2. Wells JA, O’Callaghan JM, Holmes HE, Powell NM, Johnson RA, Siow B, et al. (2015) In vivo imaging of tau pathology using multi-parametric quantitative MRI. NeuroImage 111 369–378. doi: 10.1016/j.neuroimage.2015.02.023
3. Ma D, Cardoso MJ, Zuluaga MA, Modat M, Powell N, Wiseman F, et al. (2015) Grey matter sublayer thickness estimation in the mouse cerebellum. Proc. Medical Image Computing and Computer Assisted Intervention 2015 (648).
