# Supplementary material for: Fully-Automated μMRI Morphometric Phenotyping of the Tc1 Mouse Model of Down Syndrome
Source: PLoS One. 2016 Sep 22;11(9):e0162974. doi: 10.1371/journal.pone.0162974 (PMC5033246; doi:10.1371/journal.pone.0162974)
Supplement: S3 File — This supporting information shows effect sizes at selected individual voxel locations within the TBM results. (DOCX) [file pone.0162974.s003.docx]

## S3. Individual voxel analysis of TBM results

*J_det_* values at peak absolute t-statistic locations in selected regions (indicated in Fig 10 in the main manuscript) are shown in Fig B. These help to quantify the degree of group separation in different regions. In the midbrain and hypothalamus, where the Tc1s were locally significantly larger than the WTs, the mean difference was relatively small compared with other regions. Of the regions shown, the midbrain had the lowest effect size, thanks to the relatively large standard deviation of *J_det_* values in this region, likely due to the structural variability of the fourth ventricle and aqueduct. Despite exhibiting the smallest difference between means, the peak t-statistic voxel within the hypothalamus had a moderate effect size, thanks to a low WT *J_det_* standard deviation in this region.


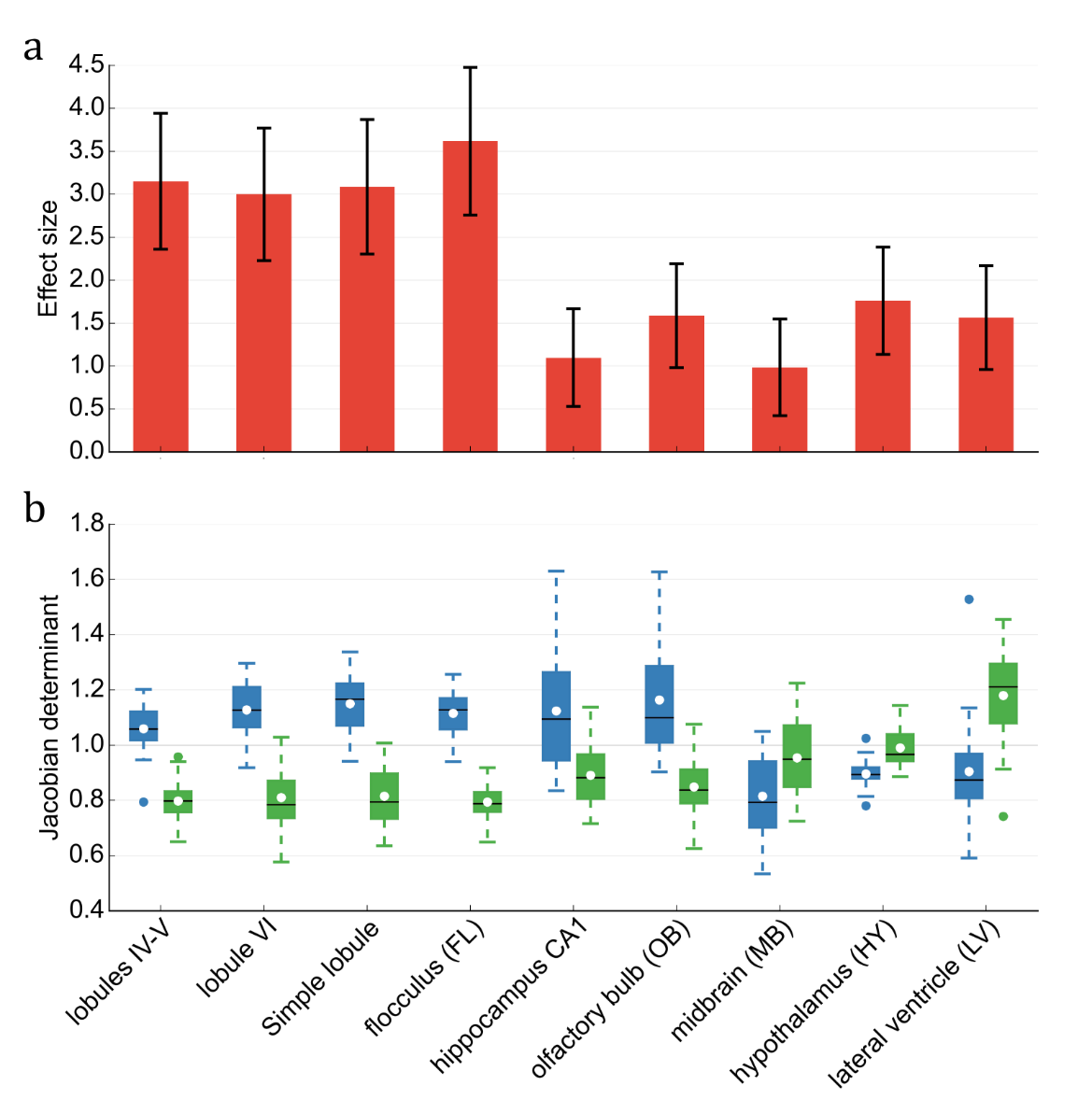


Fig B: **Peak t-statistic voxel values within selected significant clusters (from Fig 10)**. (a) Effect sizes (Cohen’s *d*) with 95% confidence intervals; (b) *J_det_* values for WT (blue) and Tc1 (green) groups. To show the effect independent of global volume, values were divided by TIV then multiplied by mean WT TIV.
