# Supplementary material for: Fully-Automated μMRI Morphometric Phenotyping of the Tc1 Mouse Model of Down Syndrome
Source: PLoS One. 2016 Sep 22;11(9):e0162974. doi: 10.1371/journal.pone.0162974 (PMC5033246; doi:10.1371/journal.pone.0162974)
Supplement: S4 File — (DOCX) [file pone.0162974.s004.docx]

## S4. Voxel-Based Morphometry results

VBM highlights local differences in GM tissue proportion, as measured by the segmentation. When performing voxel-wise statistical tests, the TIV covariate was excluded from the GLM, as the GM maps were not modulated by *J_det_* values, hence had no volume component. (With modulation, the results are dominated by the volume changes, thus are similar to TBM.)

Fig C shows representative slices through the final GWR average image, with FDR-corrected t-statistics overlaid (*q*=0.05). We smoothed with a Gaussian kernel, FWHM 0.16mm. To better anatomically localise some significant regions and improve spatial specificity, we also used a 0.02mm kernel (0.5 voxel).

VBM detected bilateral increases in GM proportion in the olfactory bulbs, thalamus, hypothalamus, midbrain, globus pallidus, motor cortex and the CA3 region of the hippocampus. The mouse midbrain, especially adjoining the pons and brainstem, exhibits a high degree of GM/WM mixture: most voxels have some PV. Additionally, this region has few high-contrast features, which may have impeded internal registration accuracy, possibly contributing to the apparent increases in GM proportion seen here. There were few significant voxels in this region after application of the narrower smoothing kernel (see Fig D), suggesting this regional apparent increase in GM proportion is sparse and nonspecific.

GM regions adjacent to the ventricles in the final average image, such as the septal nucleus, show reduced GM density in the Tc1s, likely due to ventricular expansion encroaching into GM tissue.

Bilateral regions of decreased GM density were detected in the dentate gyrus region of the hippocampus, the entorhinal cortex, and the olfactory tubercle. The cerebellum showed decreases in GM proportion throughout, particularly lobules II & III, IV-V, VI and IX, and the simple lobule. Upon inspection with a narrower smoothing kernel (Fig C), these regions appeared predominately within the granule and Purkinje cell layers.


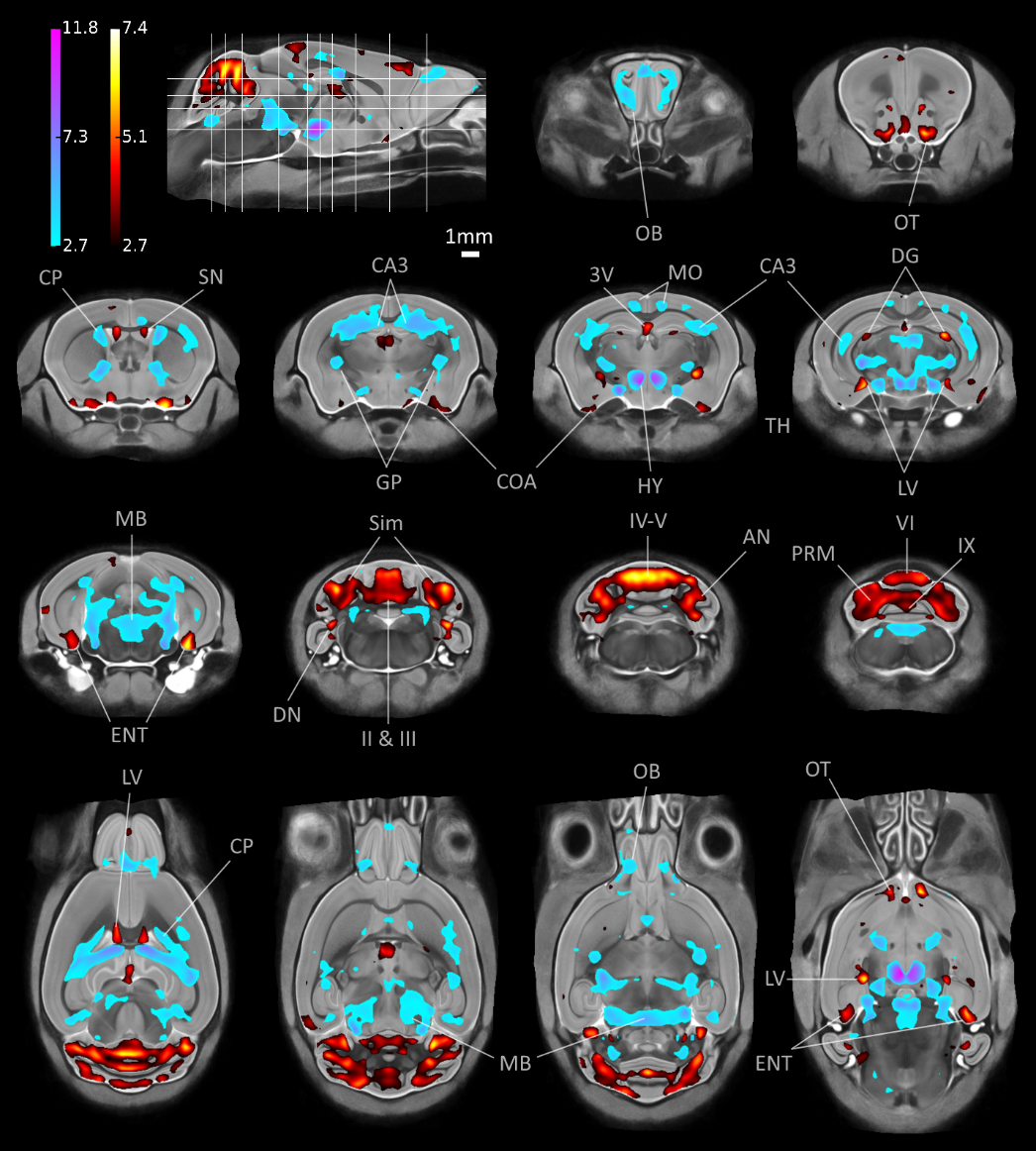


Fig C: **unmodulated** **VBM results**: FDR-corrected (*q*=0.05) t-statistics overlaid on coronal and transverse slices (locations indicated top left) of the final structural average. Blue: statistically greater local proportion of GM in Tc1 group; red: reduced. CP: caudate putamen; ENT: entorhinal cortex; GP: globus pallidus; MO: motor cortex; OT: olfactory tubercle; SN: septal nucleus. Cerebellar regions: AN: ansiform lobule; DN: dentate nucleus of the cerebellum; PRM: paramedian lobule.


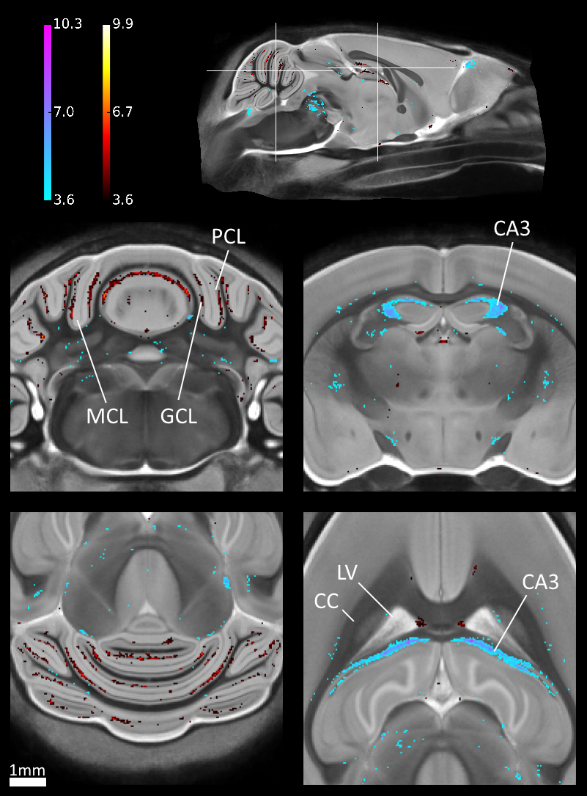


Fig D: **VBM in the cerebellum and hippocampus**: as Fig C. Sagittal, coronal and transverse views, employing a FWHM 0.02mm Gaussian smoothing kernel prior to statistical tests. Significant voxels are largely confined to the Purkinje and granule cell layers of the cerebellum and CA3 region of the hippocampus. Note few significant voxels in the midbrain. CC: corpus callosum; GCL: granule cell layer; MCL: molecular cell layer; PCL: Purkinje cell layer.
