# Supplementary material for: Fully-Automated μMRI Morphometric Phenotyping of the Tc1 Mouse Model of Down Syndrome
Source: PLoS One. 2016 Sep 22;11(9):e0162974. doi: 10.1371/journal.pone.0162974 (PMC5033246; doi:10.1371/journal.pone.0162974)
Supplement: S5 File — This supporting information includes volumes of segmented tissues and parcellations, as Table 2, from each cohort individually. (DOCX) [file pone.0162974.s005.docx]

## S5. Tissue and parcellated volumes for each cohort

We here show results from a repeat of the analysis performed for Table 2, using brains from cohort 1 (Table 3), mean (standard deviation) age 128.4 (7.4) days and cohort 2 (Table 4), age 471.5 (5.9) days separately. The pattern of mean volumes, Tc1 > WT (or vice versa) was the same, in each structure, in both cohorts, and the same as when both were combined (Table 2). The tendency when analysing the two cohorts separately was to decrease significance: p-values were generally greater and more likely to exceed the arbitrary significance threshold of p=0.05.

**Table 3**

|  | **WT** (N=14) | | **Tc1** (N=14) | | ***p*** | |
| --- | --- | --- | --- | --- | --- | --- |
|  | mean | std | mean | std | absolute | TIV-normalised |
| **a** |  |  |  |  |  |  |
| GM | 315.50 | 11.92 | 362.06 | 16.68 | 3.35x10^-8^ |  |
| WM | 116.92 | 5.55 | 131.37 | 7.16 | 1.61x10^-5^ |  |
| BV | 432.42 | 15.30 | 493.43 | 21.50 | 2.36x10^-8^ |  |
| vCSF | 2.21 | 1.09 | 2.62 | 0.61 |  |  |
| eCSF | 40.09 | 5.72 | 48.18 | 8.83 | 4.77x10^-2^ |  |
| TIV | 474.72 | 17.41 | 544.23 | 23.19 | 1.16x10^-8^ |  |
| **b** |  |  |  |  |  |  |
| amygdala | 13.52 | 0.49 | 15.49 | 0.97 | 7.88x10^-6^ |  |
| anterior commissure | 1.36 | 0.09 | 1.46 | 0.08 |  |  |
| basal forebrain and septum | 13.62 | 0.51 | 15.82 | 0.58 | 1.21x10^-9^ |  |
| brainstem | 56.86 | 1.51 | 61.10 | 2.17 | 5.61x10^-5^ | 1.48x10^-3^ |
| central GM region | 15.01 | 0.54 | 17.29 | 0.63 | 2.57x10^-9^ |  |
| cerebellum | 66.04 | 5.20 | 65.94 | 3.99 |  | 2.55x10^-5^ |
| corpus callosum & external capsule | 16.84 | 0.94 | 17.22 | 0.80 |  | 1.51x10^-3^ |
| fimbria | 3.24 | 0.35 | 3.62 | 0.26 | 0.07113 |  |
| globus pallidus | 3.97 | 0.21 | 4.46 | 0.32 | 1.05x10^-3^ |  |
| hippocampus | 29.66 | 0.94 | 31.83 | 1.33 | 7.99x10^-4^ | 0.02962 |
| hypothalamus | 12.40 | 0.59 | 14.51 | 0.66 | 4.87x10^-8^ |  |
| inferior colliculus | 7.24 | 0.61 | 8.10 | 0.38 | 2.85x10^-3^ |  |
| internal capsule | 5.21 | 0.67 | 5.06 | 0.52 |  | 0.04685 |
| midbrain (remainder) | 4.98 | 0.52 | 5.80 | 0.46 | 3.68x10^-3^ |  |
| neocortex | 142.22 | 5.98 | 153.71 | 7.12 | 2.09x10^-3^ | 0.04374 |
| olfactory bulb | 27.41 | 1.92 | 25.37 | 1.67 |  | 1.92x10^-7^ |
| striatum (caudate putamen) | 26.50 | 1.47 | 30.57 | 1.39 | 1.23x10^-6^ |  |
| superior colliculus | 9.47 | 0.81 | 11.04 | 0.98 | 2.14x10^-3^ |  |
| thalamus | 26.33 | 1.10 | 27.81 | 1.76 |  | 0.01597 |
| ventricles | 1.65 | 0.22 | 1.92 | 0.31 |  |  |

Cohort 1 mean absolute volumes (mm^3^), by group, of (a) probabilistic tissues: $BV=GM+WM$; $TIV=BV+CSF$ and (b) parcellated regions via integration of Jacobian determinants, and their standard deviations. (Bonferroni-adjusted two-tailed p-values shown, omitted where >>0.05).

**Table 4**

|  | **WT** (N=12) | | **Tc1** (N=15) | | ***p*** | |
| --- | --- | --- | --- | --- | --- | --- |
|  | mean | std | mean | std | absolute | TIV-normalised |
| **a** |  |  |  |  |  |  |
| GM | 317.69 | 6.31 | 364.52 | 24.96 | 7.82x10^-6^ |  |
| WM | 132.03 | 5.97 | 147.23 | 8.78 | 1.64x10^-4^ |  |
| BV | 449.71 | 9.61 | 511.75 | 32.26 | 6.14x10^-6^ |  |
| vCSF | 2.60 | 0.83 | 4.18 | 1.24 | 5.02x10^-3^ | 5.05x10^-2^ |
| eCSF | 11.09 | 4.52 | 12.74 | 3.69 |  |  |
| TIV | 463.39 | 10.06 | 528.68 | 31.32 | 1.79x10^-6^ |  |
| **b** |  |  |  |  |  |  |
| amygdala | 11.33 | 0.31 | 13.68 | 0.70 | 1.28x10^-9^ | 0.02939 |
| anterior commissure | 1.58 | 0.06 | 1.69 | 0.08 | 0.0133 | 0.03138 |
| basal forebrain and septum | 13.19 | 0.37 | 15.75 | 0.61 | 4.78x10^-11^ |  |
| brainstem | 56.13 | 2.14 | 59.13 | 2.72 |  | 0.01341 |
| central GM region | 15.68 | 0.27 | 17.49 | 0.68 | 1.13x10^-7^ |  |
| cerebellum | 62.06 | 2.58 | 57.77 | 3.65 | 0.0465 | 3.73x10^-8^ |
| corpus callosum & external capsule | 18.70 | 0.39 | 20.43 | 1.18 | 1.25x10^-3^ |  |
| fimbria | 3.91 | 0.18 | 4.45 | 0.26 | 3.51x10^-5^ |  |
| globus pallidus | 4.54 | 0.13 | 4.66 | 0.25 |  | 4.37x10^-7^ |
| hippocampus | 28.43 | 0.64 | 31.08 | 1.32 | 2.49x10^-5^ | 0.01491 |
| hypothalamus | 12.09 | 0.27 | 14.10 | 0.49 | 5.63x10^-11^ |  |
| inferior colliculus | 7.80 | 0.34 | 8.15 | 0.45 |  | 5.48x10^-3^ |
| internal capsule | 5.79 | 0.29 | 5.72 | 0.50 |  | 1.00x10^-3^ |
| midbrain (remainder) | 4.95 | 0.19 | 5.65 | 0.27 | 1.49x10^-6^ |  |
| neocortex | 128.79 | 1.86 | 138.38 | 4.36 | 4.43x10^-6^ | 7.05x10^-3^ |
| olfactory bulb | 25.17 | 0.69 | 25.02 | 1.27 |  | 3.68x10^-7^ |
| striatum (caudate putamen) | 27.23 | 0.64 | 30.86 | 1.66 | 4.20x10^-6^ |  |
| superior colliculus | 9.24 | 0.27 | 10.91 | 0.61 | 8.68x10^-8^ |  |
| thalamus | 26.56 | 0.50 | 27.42 | 1.13 |  | 3.27x10^-7^ |
| ventricles | 1.65 | 0.24 | 2.50 | 0.38 | 1.30x10^-5^ | 1.62x10^-3^ |

Cohort 2 mean absolute volumes (mm^3^), by group, of (a) probabilistic tissues: $BV=GM+WM$; $TIV=BV+CSF$ and (b) parcellated regions via integration of Jacobian determinants, and their standard deviations. (Bonferroni-adjusted two-tailed p-values shown, omitted where >>0.05).
